# Randomized multicentre pilot study of sacubitril/valsartan versus irbesartan in patients with chronic kidney disease: United Kingdom Heart and Renal Protection (HARP)- III—rationale, trial design and baseline data

**DOI:** 10.1093/ndt/gfw321

**Published:** 2016-09-17

**Authors:** UK HARP-III Collaborative Group, PK Judge, R Haynes, WG Herrington, BC Storey, N Staplin, A Bethel, L Bowman, N Brunskill, P Cockwell, R Dayanandan, M Hill, PA Kalra, JJ McMurray, M Taal, DC Wheeler, MJ Landray, C Baigent, C Baigent, R Haynes, MJ Landray, R Dayanandan, A Baxter, N Staplin, A Bethel, L Bowman, N Brunskill, P Cockwell, WG Herrington, M Hill, PK Judge, PA Kalra, C Knott, JJ McMurray, K Murphy, M Taal, DC Wheeler, K Wheatley, J Emberson, C Tomson, P Roderick

**Keywords:** cardiovascular disease, chronic kidney disease, neprilysin, progression

## Abstract

**Background:**

Patients with chronic kidney disease (CKD) are at risk of progression to end-stage renal disease and cardiovascular disease. Data from other populations and animal experiments suggest that neprilysin inhibition (which augments the natriuretic peptide system) may reduce these risks, but clinical trials among patients with CKD are required to test this hypothesis.

**Methods:**

UK Heart and Renal Protection III (HARP-III) is a multicentre, double-blind, randomized controlled trial comparing sacubitril/valsartan 97/103 mg two times daily (an angiotensin receptor–neprilysin inhibitor) with irbesartan 300 mg one time daily among 414 patients with CKD. Patients ≥18 years of age with an estimated glomerular filtration rate (eGFR) of ≥45 but <60 mL/min/1.73 m^2^ and urine albumin:creatinine ratio (uACR) >20 mg/mmol or eGFR ≥20 but <45 mL/min/1.73 m^2^ (regardless of uACR) were invited to be screened. Following a 4- to 7-week pre-randomization single-blind placebo run-in phase (during which any current renin–angiotensin system inhibitors were stopped), willing and eligible participants were randomly assigned either sacubitril/valsartan or irbesartan and followed-up for 12 months. The primary aim was to compare the effects of sacubitril/valsartan and irbesartan on measured GFR after 12 months of therapy. Important secondary outcomes include effects on albuminuria, change in eGFR over time and the safety and tolerability of sacubitril/valsartan in CKD.

**Results:**

Between November 2014 and January 2016, 620 patients attended a screening visit and 566 (91%) entered the pre-randomization run-in phase. Of these, 414 (73%) participants were randomized (mean age 63 years; 72% male). The mean eGFR was 34.0 mL/min/1.73 m^2^ and the median uACR was 58.5 mg/mmol.

**Conclusions:**

UK HARP-III will provide important information on the short-term effects of sacubitril/valsartan on renal function, tolerability and safety among patients with CKD.

## INTRODUCTION

Chronic kidney disease (CKD) affects between 2 and 17% of the general population (depending on the country) [1, 2] and is associated with increased risks of progression to end-stage renal disease (ESRD) and morbidity and mortality from cardiovascular disease (CVD) [3, 4]. Renin–angiotensin system (RAS) inhibitors [angiotensin-converting enzyme inhibitors (ACEis) and angiotensin receptor blockers (ARBs)] have been shown to reduce the risk of ESRD in patients with proteinuric CKD [5–8], but despite such treatments, patients remain at significant risk of progression to ESRD and CVD.

The natriuretic peptide (NP) system is a neurohormonal system that has a variety of potentially beneficial functions, including natriuresis, diuresis, vasodilatation and counterregulation of RAS [9, 10]. The NP system can be augmented by inhibiting the main enzyme responsible for degrading NPs, namely neprilysin [or neutral endopeptidase (NEP)] [10]. NEP is a membrane-bound zinc-containing metalloproteinase [11] that also degrades other peptides, including angiotensin II, bradykinin, endothelin and substance P [12]. However, isolated NEP inhibition (NEPi) leads to reflex RAS activation, and inhibits angiotensin II breakdown (counteracting any potentially beneficial effects) and therefore NEPi must be combined with RAS inhibition.

As NEPi and ACEi both inhibit bradykinin degradation, their combination is associated with substantially elevated bradykinin levels that cause unacceptable rates of angioedema [13]. ARBs do not inhibit bradykinin degradation and can be safely combined with NEPi [creating a new class of drugs called angiotensin receptor–neprilysin inhibitors (ARNis)]. Sacubitril/valsartan (previously known as LCZ696) is the first drug in this new class, combining valsartan with sacubitril [(AHU377) a prodrug that is metabolized via esterases to the active NEPi sacubitrilat (LBQ657)]. Sacubitril/valsartan 97/103 mg provides equivalent plasma concentrations of valsartan as oral valsartan 160 mg [14].

In a 5/6 nephrectomy model, treatment with combined NEP/RAS inhibition was associated with greater reductions in proteinuria and glomerulosclerosis compared with RAS inhibition alone [15, 16]. Micropuncture studies also demonstrated NEPi led to greater reductions in capillary glomerular pressure [15]. Among patients with heart failure, trials comparing sacubitril/valsartan with either ACEi or ARB have suggested that the estimated glomerular filtration rate (eGFR) of patients allocated sacubitril/valsartan declined less than those assigned ACEi or ARB [17, 18]. Sacubitril/valsartan also reduced blood pressure more than equivalent doses of valsartan in trials among patients with elevated blood pressure [19]. Trials in heart failure populations suggest NEPi might increase albuminuria [18, 20], but this effect was not observed in patients with hypertension [19] and baseline albuminuria was very low in all these trials. Overall, these data raise the hypothesis that treatment with an ARNi may be superior to either ACEi or ARB alone in slowing the progression of CKD.

The United Kingdom (UK) Heart and Renal Protection III(HARP-III) trial (ISRCTN11958993) was designed to provide information on the short-term efficacy (in terms of effect on renal function), tolerability and safety of sacubitril/valsartan among patients with CKD. The trial will also assess the effects of sacubitril/valsartan on albuminuria, blood pressure and biomarkers of kidney and cardiac damage.

## MATERIALS AND METHODS

### Study design

**FIGURE 1:**
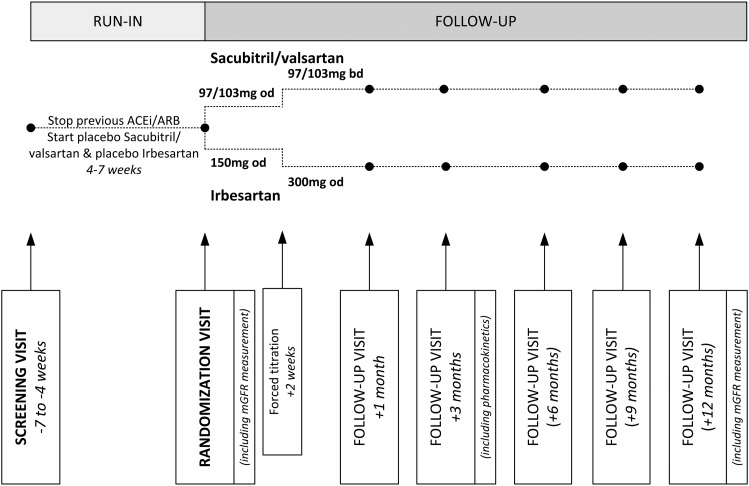
UK HARP-III trial design.

**FIGURE 2:**
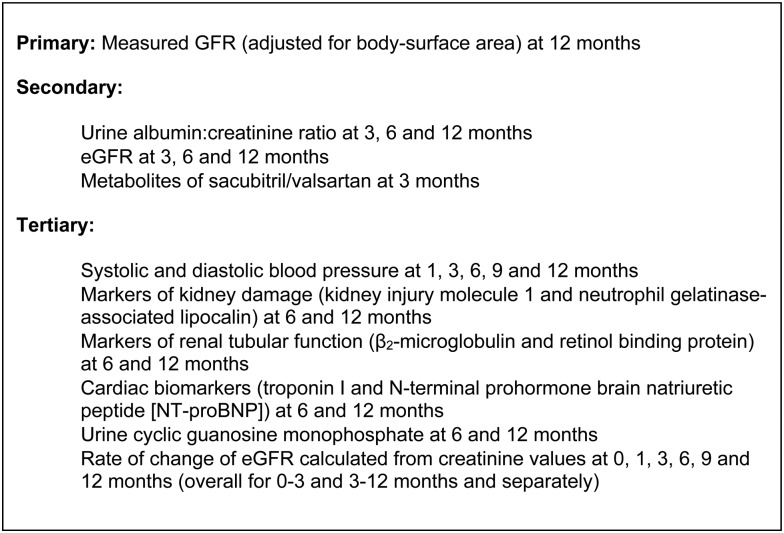
UK HARP-III trial outcomes.

UK HARP-III is a double-blind, multicentre, randomized controlled trial comparing sacubitril/valsartan 97/103 mg two times daily versus irbesartan 300 mg one time daily among at least 400 participants ≥18 years of age with stages 3 and 4 CKD. Irbesartan 300 mg was selected as the comparator, as it has been shown to reduce the risk of ESRD among patients with diabetic kidney disease and is licensed for the treatment of proteinuric CKD [6, 21]. Participants were randomly allocated to receive sacubitril/valsartan or irbesartan and will be followed up for 1 year (Figure 1). The primary aim of UK HARP-III is to assess the effect of sacubitril/valsartan 97/103 mg two times daily versus irbesartan 300 mg one time daily on measured glomerular filtration rate (mGFR) at 12 months. Important secondary outcomes include the effect on urine albumin:creatinine ratio (uACR) and eGFR. All the secondary and tertiary assessments are shown in Figure 2 and further details are available in the data analysis plan (see Supplementary data). A summary of substantial amendments to the protocol is provided in the Supplementary data.

### Eligibility

**FIGURE 3:**
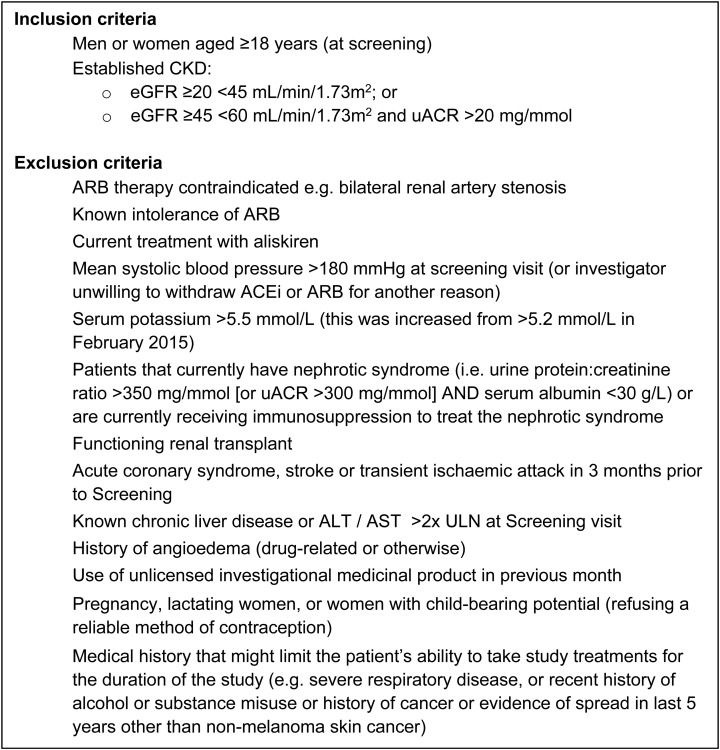
Inclusion and exclusion criteria. ALT, alanine aminotransferase; AST, aspartate aminotransferase; ULN, upper limit of normal.

To fulfil the inclusion criteria, patients need to be ≥18 years of age and have either an eGFR ≥45 but <60 mL/min/1.73 m^2^ with a uACR >20 mg/mmol or eGFR ≥20 but <45 mL/min/1.73 m^2^ (regardless of uACR). The exclusion criteria were designed to identify patients for whom the safety of sacubitril/valsartan or irbesartan may have been a concern. The full eligibility criteria are shown in Figure 3.

### Study enrolment and randomization

#### Identification and invitation

After relevant ethics [Nottingham Research Ethics Committee 2 (13/EM/0434)] and regulatory approvals had been obtained, sites were established in UK renal units. Site staff identified potentially eligible patients from hospital electronic databases, mailed these individuals an invitation letter and a copy of the patient information sheet and called them ∼1 week later to discuss the trial in more detail, answer any questions they might have and to see whether they were interested in participating. Those individuals interested in participating were invited to attend a screening visit.

#### Screening

At the screening visit, eligibility was assessed and written informed consent was obtained from eligible individuals. All data were recorded directly into a bespoke Internet-based electronic case report form system. Relevant details of their medical history (including primary renal diagnosis, presence of diabetes mellitus and prior CVD) were recorded by trained research nurses and their height, weight and blood pressure were measured. Blood pressure was measured and recorded three times using an Omron M6 automated digital sphygmomanometer after sitting for at least 5 minutes. Willing and eligible patients entered the pre-randomization run-in phase. Samples of blood and urine were sent to the local hospital laboratory for confirmation of eligibility. If the results were considered inaccurate (e.g. haemolysed sample) by the local study staff the samples could be repeated once, but if the results did not confirm eligibility the participant was withdrawn from the run-in phase.

#### Pre-randomization run-in

The aims of the pre-randomization run-in phase were (i) to ‘wash out’ any ACEi prior to introduction of NEPi, (ii) to allow a comparison of the acute effects of the study treatments on GFR and (iii) to reduce the rate of post-randomization discontinuation of study treatment and to produce a consequent improvement in the trial's statistical sensitivity [22]. Following the screening visit, any current ACEi and/or ARB that the participant was taking was stopped and the participant entered the 4- to 7-week single-blind pre-randomization run-in phase, during which they were asked to take one placebo sacubitril/valsartan tablet and one placebo irbesartan capsule once daily. If elevated blood pressure became a concern during the run-in phase, local investigators were advised to titrate up or start additional anti-hypertensive medications, but to avoid an ACEi, ARB or direct renin inhibitor (DRI). The choice of additional anti-hypertensive therapy remained at the discretion of the responsible clinician. Participants could withdraw from the trial for any reason during this run-in phase. Participants who did not withdraw returned 4–7 weeks later and had their GFR measured and attended a randomization visit. GFR was measured using a standard ^51^Cr-EDTA technique, although if this was not available at the site, other methods (^99m^Tc-DTPA or iohexol) could be used with the agreement of the coordinating centre. In willing participants, a 24-hour collection of urine for albumin and sodium quantification was also obtained.

#### Randomization visit

Participants were not eligible for randomization if the mean of their second and third measurements of systolic blood pressure was <110 mmHg (or <130 mmHg with symptoms of hypotension) or if they reported an adverse event they believed to be related to their run-in treatment. Participants who remained willing and eligible were then randomly allocated in a 1:1 ratio to receive either sacubitril/valsartan or irbesartan. Participants were randomized by an Internet-based system using a minimization algorithm to ensure balance of important predictors of renal progression, including age, sex, systolic blood pressure, eGFR, uACR and the presence or absence of diabetes mellitus.

At the randomization visit, run-in treatment was collected and willing and eligible participants were issued two bottles of study treatments: one containing sacubitril/valsartan 97/103 mg or placebo tablets and the other containing irbesartan 150 mg or placebo capsules (therefore a double-dummy technique to protect blinding). Participants were initially instructed to take one tablet and one capsule daily in the morning (i.e. either sacubitril/valsartan 97/103 mg plus placebo irbesartan or placebo sacubitril/valsartan plus irbesartan 150 mg). Blood and urine samples were collected for the local analysis of creatinine, electrolytes, liver function tests and uACR and others were prepared for central analysis (Table 1).

**Table 1:** Planned central laboratory blood and urine analyses

Analyte	Time point			
	Randomization	3 months	6 months	12 months
EDTA plasma samples				
Creatinine	▪	▪	▪	▪
Albumin	▪		▪	▪
Troponin-I	▪		▪	▪
NT-proBNP	▪		▪	▪
CRP	▪		▪	▪
IL-6	▪		▪	▪
*Urine samples*				
Albumin:creatinine ratio	▪	▪	▪	▪
KIM-1	▪		▪	▪
NGAL	▪		▪	▪
cGMP	▪		▪	▪
β-2-microglobulin	▪		▪	▪
Retinol binding protein	▪		▪	▪

NT-proBNP, N-terminal prohormone brain natriuretic peptide; CRP, C-reactive protein; IL-6, interleukin 6; KIM-1, kidney injury molecule 1; NGAL, neutrophil gelatinase-associated lipocalin; cGMP, cyclic guanosine monophosphate.

### Post-randomization follow-up

Randomization is now complete and all participants are in follow-up. In order to check potassium and renal function after starting study treatment, participants attend their study clinic or local primary care physician at 2 weeks after randomization for a blood sample. If these results are satisfactory, study treatments are increased to either sacubitril/valsartan 97/103 mg two times daily plus two capsules of placebo irbesartan one time daily or one tablet of placebo sacubitril/valsartan two times daily plus irbesartan 300 mg one time daily.

#### Follow-up assessments

Study follow-up visits are scheduled at 1, 3, 6, 9 and 12 months after randomization. At all visits, study staff systematically seek the information on all serious adverse events, any non-serious adverse events considered by participants to be related to study treatment; and on symptoms of hepatitis. Compliance with study treatment is assessed and participants unable to tolerate the maximum dose of study treatments are encouraged to continue on the lower dose of study drug (i.e. sacubitril/valsartan 97/103 mg or irbesartan 150 mg daily) for the remainder of the trial. If relevant, a reason for discontinuation or dose reduction is recorded. Participants prescribed contraindicated medications (ACEi, ARB or DRI) have their randomized treatment stopped. Weight and blood pressure are measured (three times after sitting for at least 5 minutes) at all visits. In both treatment groups, blood pressure is to be controlled according to the Kidney Disease: Improving Global Outcomes guidelines [23], with the initiation and choice of additional anti-hypertensive treatment being at the discretion of the responsible clinician. Within the 2 weeks before their 12-month visit participants have their second GFR measurement (using the same method as at baseline). Copies of results of both measurements of GFR are sent to the coordinating centre so the results entered by site staff can be verified by clinical study staff blind to the treatment allocation.

#### Biological samples and safety monitoring

At each follow-up visit, blood and urine samples are sent to the local hospital laboratory for creatinine, electrolytes, liver function tests (bilirubin, alanine or aspartate transaminase and alkaline phosphatase) and uACR. In addition, at the 3-, 6- and 12-month visits, samples are also taken for central analysis. EDTA samples are centrifuged and the plasma aliquoted into Cryovials, which are stored locally (with Cryovials of urine) at or below −20°C prior to transfer to the central laboratory in Oxford, UK, where they are stored at −80°C. The main plasma analytes measured at the central laboratory are creatinine, cardiac and inflammatory biomarkers and the urine analytes include albumin and markers of tubular damage and function [including kidney injury molecule 1, neutrophil gelatinase-associated lipocalin, β2-microglobulin and retinol binding protein; Table 1]. Participants are asked not to take their morning dose of study treatment on the day of their 3-month visit (at this visit only) and the date and time of the last dose is recorded, as these samples are to be used for pharmacokinetic analyses.

The results of local samples are entered into the trial database once available and reviewed daily by a trained clinician at the coordinating centre. If the potassium is >5.5 mmol/L, alanine or aspartate transaminase >2× the upper limit of normal or if the eGFR has fallen >25% from the previous value, then the trial protocol provides advice on further tests and study treatment (see Supplementary data).

### Monitoring

Prior to starting recruitment, study staff received training in the study procedures and the web-based data collection system at the coordinating centre. Recruitment rates, adherence to trial procedures and completeness of follow-up data are monitored closely by staff at the coordinating centre. All sites have at least one on-site monitoring visit, with further visits as indicated by the results of central monitoring of the data. An independent data monitoring committee (see Supplementary data) regularly reviews unblinded interim analyses of all relevant data.

### Statistical considerations

#### Sample size

The chief aim of this study is to compare mGFR between the two treatment groups at the final follow-up visit. Analysis of covariance (ANCOVA) compares mean follow-up mGFR between treatment groups after adjustment for baseline mGFR [24]. Assuming a between-person standard deviation (SD) in mGFR of 15 mL/min/1.73 m^2^ and a correlation between an individual's baseline and follow-up mGFR of 0.8, randomization of 400 participants will provide at least 80% power (at 2 P = 0.05) to detect a difference in mGFR at the final follow-up (adjusted for baseline values) of 3 mL/min/1.73 m^2^ (the chosen minimum clinically meaningful difference), even if 15% of participants discontinue allocated study treatment [20].

#### Statistical analysis

All analyses will involve comparing outcomes during the scheduled treatment period among all those participants allocated at randomization to receive sacubitril/valsartan 97/103 mg two times daily versus all those allocated to receive irbesartan 300 mg one time daily [i.e. intention-to-treat (ITT) analyses] [25, 26]. Comparisons of continuous outcomes (including the primary outcome) between the allocated treatment arms will be performed using ANCOVA adjusted for each patient's value at baseline [27]. If continuous outcomes are not normally distributed, then appropriate transformations (e.g. log transformation) will be made. Multiple imputation techniques will be used to account for any missing data in the primary and secondary outcomes [28]. Further details are provided in the data analysis plan (see Supplementary data).

## RESULTS

**FIGURE 4:**
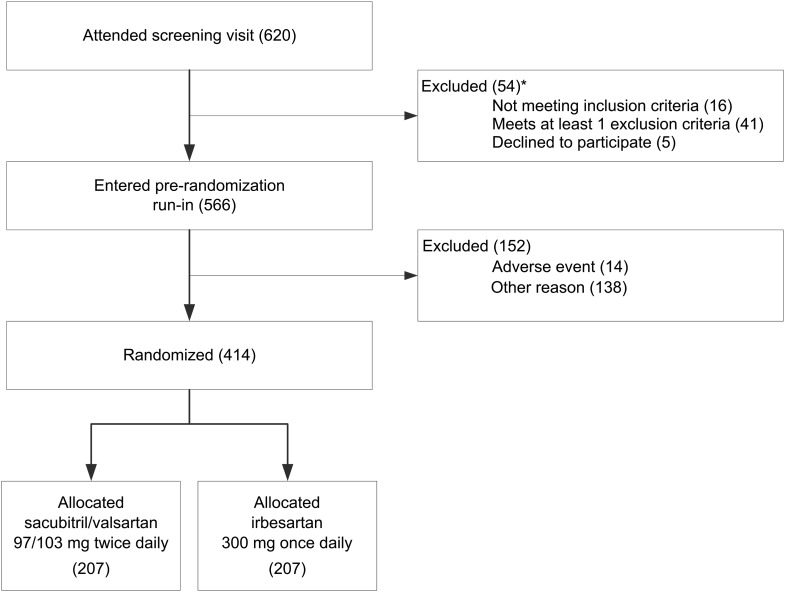
Trial profile: flow of participants through the trial. *Indicates that participants may have more than one reason.

Study sites were established in 24 renal units in the UK. Between November 2014 and January 2016 a total of 620 patients attended the study screening visits and 566 (91%) entered the pre-randomization run-in (Figure 4).

### Pre-randomization run-in

A total of 138 participants withdrew from the pre-randomization run-in before attending a randomization visit (Table 2A). The most common medical reason for withdrawal from run-in was that the results from blood and urine samples taken at the screening visit did not confirm the participant's eligibility (Table 2A). Adverse events were uncommon and four participants were withdrawn because of a serious adverse event (myocardial infarction, septic shock and two cases of pneumonia).

**Table 2:** Reasons for (A) withdrawal during run-in and (B) ineligibility at a randomization visit

(A)	*n* (%)
Number entering run-in	566
*Adverse event*	
Serious adverse event	4 (3)
Non-serious adverse reaction	7 (5)
*Other reason*	
Ineligible on laboratory results sent at screening visit	59 (43)
Participant wishes	16 (12)
Medical advice	13 (9)
Other non-medical reason	39 (28)
Total withdrawn during Run-in	138 (100)
(B)	
Number attending randomization visit	428
*Adverse event*	
Serious adverse event	0 (0)
Non-serious adverse reaction	3 (21)
*Other reason*	
Blood pressure too low	9 (64)
Other	2 (14)
Total ineligible at randomization visit	14 (100)

In addition, 14 individuals attended a randomization visit but were not eligible to be randomized: the most common reason for this was their blood pressure being too low (Table 2B). Overall, 152 (27%) of the 566 individuals who entered the pre-randomization single-blind placebo run-in phase were not subsequently randomized.

### Baseline characteristics of randomized participants

A total of 414 people were randomized (Figure 4). The mean age was 63 (SD 14) years and 298 (72%) were male (Table 3). The mean systolic blood pressure was 146 (SD 16) mmHg at randomization (i.e. after 4–7 weeks of withdrawal of any prior ACEi or ARB). Based on results from the local laboratories, the mean eGFR was 34.0 (SD 10.6) mL/min/1.73 m^2^ and the median uACR was 58.5 (interquartile range 12.5–156.3) mg/mmol. Central laboratory assays will be conducted at the end of the study. About half of randomized participants had either glomerular [111 (27%)] or diabetic [83 (20%)] kidney disease and 165 (40%) patients reported diabetes mellitus at baseline. The median 5-year risk of ESRD (calculated using a validated risk calculator [29] was 16.5%, and 62% of participants had a 5-year risk >10%.

**Table 3:** Baseline characteristics of UK HARP-III participants

Baseline characteristic	All participants (*n* = 414)
Age (years)	63 ± 14
<50	73 (18)
≥50–70	196 (47)
≥70	145 (35)
Gender	
Male	298 (72)
Female	116 (28)
Ethnicity	
White	377 (91)
Black	7 (2)
South Asian	18 (4)
Other	12 (3)
Prior disease	
Coronary heart disease	55 (13)
Cerebrovascular disease	31 (7)
Peripheral arterial disease	44 (11)
Heart failure	17 (4)
Diabetes	165 (40)
Systolic blood pressure (mmHg)	146 ± 16
<140	149 (36)
≥140–160	180 (43)
≥160	85 (21)
Diastolic blood pressure (mmHg)	81 ± 11
<80	191 (46)
≥80–90	133 (32)
≥90	90 (22)
Body mass index (kg/m^2^)	30.6 ± 6.2
<25	68 (16)
≥25–30	147 (36)
≥30	195 (47)
Not available	4 (1)
Medication	
Antiplatelet therapy	138 (33)
Oral anticoagulant	28 (7)
Diuretic	164 (40)
Calcium channel blocker	207 (50)
β-blocker	112 (27)
α-blocker	112 (27)
LDL-lowering agent	263 (64)
Prior use of ACEi or ARB	
Yes	339 (82)
No	75 (18)
CKD-EPI eGFR (mL/min/1.73 m^2^)	34.0 ± 10.6
<30	169 (41)
≥30–45	176 (43)
≥45	64 (15)
Not available	5 (1)
Urine albumin:creatinine ratio (mg/mmol)	58.5 (12.5-156.3)
<3	48 (12)
≥3–< 30	88 (21)
≥30	251 (61)
Not available	27 (7)
Primary renal diagnosis	
Glomerular disease	111 (27)
Tubulointerstitial disease	50 (12)
Diabetic kidney disease	83 (20)
Hypertensive/renovascular disease	42 (10)
Other systemic diseases affecting the kidneys	3 (1)
Familial/hereditary nephropathies	43 (10)
Miscellaneous renal disorders	9 (2)
Unknown	73 (18)

Recorded at randomization visit unless otherwise stated. Values are given as *n* (%), mean ± SD or median (interquartile range).

CKD-EPI, Chronic Kidney Disease Epidemiology Collaboration; LDL, low-density lipoprotein.

## DISCUSSION

The UK HARP-III trial has recruited 414 participants with CKD and will provide information on the short-term effects of sacubitril/valsartan on the change in kidney function (using mGFR) and the tolerability and safety of the drug compared with irbesartan in people with CKD. The trial will also provide information on the effects of sacubitril/valsartan on albuminuria, blood pressure and other biomarkers of both kidney and cardiac function. These results are important because sacubitril/valsartan has now entered routine clinical practice as a treatment for heart failure with reduced ejection fraction (HFrEF) [30], and many of these patients also have CKD. Moreover, NEPi has the potential to be a useful treatment for CKD itself.

Large randomized trials of interventions to slow the progression of CKD are required since currently available treatments do not prevent ESRD in all patients with CKD. Although ACEis and ARBs reduce the risk of progression of proteinuric diabetic and non-diabetic kidney disease, their effect (like most medical treatments) is moderate. For example, in proteinuric diabetic kidney disease, irbesartan reduced the risk of ESRD, doubling of creatinine or death from any cause by 20% compared with placebo {hazard ratio [HR] 0.80 [95% confidence interval (CI) 0.66–0.97]; P = 0.02}, but this composite outcome still occurred in nearly one-third of those allocated irbesartan (and 14% reached ESRD) during the mean 2.6 years of follow-up [6]. Other strategies to reduce the risk of renal progression have either been ineffective, hazardous or both [31–33]. Neprilysin inhibition appears to be effective in rat models of CKD [15, 16, 34], but these are poorly predictive of efficacy in humans [35, 36]. In addition, sacubitril/valsartan has been shown to increase albuminuria in trials among patients with heart failure (who typically have very low baseline albuminuria) [18, 20]. NPs (particularly atrial NP) cause afferent arteriolar vasodilatation [37, 38] that may lead to increased intraglomerular pressure and hyperfiltration, which would be detrimental to the kidney. However, NEPi also disturbs degradation of other vasoactive peptides, so the net effect of NEPi on glomerular haemodynamics is uncertain, and in rat models at least, it appears to be favourable [15, 16, 34]. NPs may alter glomerular permeability and/or tubular reabsorption of protein, which may lead to albuminuria without hyperfiltration, the consequences of which are uncertain. UK HARP-III is the first trial of NEPi in humans with CKD and the measurements of GFR, albuminuria and other markers of kidney function and damage will help to resolve these uncertainties.

Most patients with CKD do not progress to ESRD [39], but are at high risk of CVD [4]. Lowering low-density lipoprotein cholesterol has been shown to clearly reduce the risk of atherosclerotic vascular disease in CKD [40]. However, as renal function declines, the pattern of CVD changes from atherosclerotic disease (i.e. myocardial infarction, ischaemic stroke) to non-atherosclerotic disease (characterized by arteriosclerosis and structural heart disease, which manifests clinically similarly to heart failure, with a high incidence of sudden cardiac death) [4, 41–43], but effective treatments for non-atherosclerotic disease are not yet available. Lowering blood pressure in patients with CKD appears to reduce the risk of a wide variety of cardiovascular events, but residual risk remains [44]. The similarities in the manifestation of non-atherosclerotic disease observed in CKD and heart failure suggest that treatments that are effective in heart failure may well also be effective at reducing cardiovascular risk among patients with CKD. In the Prospective Comparison of ARNI with ACEI to Determine Impact on Global Mortality and Morbidity in Heart Failure (PARADIGM-HF) trial, sacubitril/valsartan reduced the risk of cardiovascular mortality or hospitalization for heart failure by 20% [HR 0.80 (95% CI 0.73–0.93) P < 0.001] compared with enalapril, with similar effects observed among participants with and without CKD at baseline [45]. These data suggest that NEPi would be an ideal candidate to test among patients with CKD. Nevertheless, most patients with CKD have a normal ejection fraction [41, 43], and treatments that improve outcomes in HFrEF do not necessarily improve outcomes in patients with heart failure with preserved ejection fraction [46, 47], so direct evidence is needed. NEPi has improved cardiac biomarkers (e.g. troponin, N-terminal prohormone brain NP in trials in heart failure [18, 48], so the effects of NEPi on these cardiac biomarkers in people with CKD will also be of interest.

NEPi has the potential to improve both renal and cardiovascular outcomes among patients with CKD. The UK HARP-III trial will provide important information on the efficacy, safety and tolerability of sacubitril/valsartan in people with CKD. Results are anticipated in 2017.

## SUPPLEMENTARY DATA

Supplementary data are available online at http://ndt.oxfordjournals.org.

## Supplementary Material

Supplementary Data

## WRITING COMMITTEE

P.K. Judge*, R. Haynes*, W.G. Herrington, B.C. Storey, N. Staplin, A. Bethel, L. Bowman, N. Brunskill, P. Cockwell, R. Dayanandan, M. Hill, P.A. Kalra, J.J. McMurray, M. Taal, D.C. Wheeler, M.J. Landray^†^, C. Baigent^†^

*,^†^ Joint contribution.

## STEERING COMMITTEE

*Chairman* C Baigent *Principal Investigators* R Haynes (Clinical Coordinator), MJ Landray *Administrative coordinator* R Dayanandan *Computing* A Baxter *Statistics* N Staplin *Other members* A Bethel, L Bowman, N Brunskill, P Cockwell, WG Herrington, M Hill, PK Judge, PA Kalra, C Knott, JJ McMurray, K Murphy, M Taal, DC Wheeler.

## DATA MONITORING COMMITTEE

*Chairman* K Wheatley *Statistician* J Emberson *Other members* C Tomson, P Roderick
