# Supplementary material for: Randomized multicentre pilot study of sacubitril/valsartan versus irbesartan in patients with chronic kidney disease: United Kingdom Heart and Renal Protection (HARP)- III—rationale, trial design and baseline data
Source: Nephrol Dial Transplant. 2016 Sep 17;32(12):2043–51. doi: 10.1093/ndt/gfw321 (PMC5837485; doi:10.1093/ndt/gfw321)
Supplement: Supplementary Data [file gfw321_uk_harp-iii_baseline_paper_supp_appendix_submitted.docx]

**Randomized multicentre pilot study of sabubitril/valsartan versus irbesartan in patients with chronic kidney disease: UK Heart and Renal Protection (UK HARP)-III.**

**Rationale, trial design and baseline data**

**SUPPLEMENTARY APPENDIX**

|  | Page |
| --- | --- |
| Collaborators and committees | 2 |
|  |  |
| Data Analysis Plan | 4 |
|  |  |
| Safety monitoring procedures | 17 |
|  |  |
| Substantial amendments to study protocol | 19 |

***Coordinating centre and collaborators***

**Central Coordinating Centre (Clinical Trial Service Unit, University of Oxford):** *Administration and support* R Dayanandan (coordinator), P Achiri, A Burke, L Cureton, R Davis, S Fathers, L Fletcher, K Frederick, J Heineman, K Murphy, L Pank, A Panicker, E Pearson-Burton, S Pickworth, Y Qiao, A Radley, J Sayer, S Shah, A Timadjer, M Willett, K Vandenberg; *Clinical support* R Haynes (coordinator), WG Herrington, PK Judge, M Mafham, BC Storey; *Computing coordinators* A Baxter, P Dalton, R Goodenough, M Lay, *Computing* R Ait-Sadi, I Barton, G Blower, C Daniels, K Jayne, D Lukasz, A Maskill, A Murawska; *CTSU Wolfson Laboratory* M Hill (coordinator), K Chung, C Guest, A Kumar, N Luker, M Radley, J Taylor, L Weaving, J Wintour, M Yeung; *Nurse monitors* C Knott (coordinator), J Chambers, D Donaldson, J Henderson, H Lochhead, E Walton, A Wilson; *Statistical analysis* N Staplin, W Stevens.

**Collaborators**

*Aberdeen Royal Infirmary:* L Clark, J Annand, A Marks, H Rose; *Derby Teaching Hospitals NHS Foundation Trust:* M Taal, T Brear, L Havill, J McKinnell, S Melbourne, A Shardlow, J Smith, C Stone; *Derriford Hospital, Plymouth Hospitals NHS Trust:* P Rowe, A Connor, J Wooding; *Dorset County Hospital NHS Foundation Trust:* J Taylor, L Bough, K Purse, S Wignall, B Winter-Goodwin; *Leicester General Hospital, University Hospitals of Leicester NHS Trust*: N Brunskill, B Bailey, J Barratt, E Bielecka, M McCartney; *Manchester Royal Infirmary, Central Manchester University Hospitals NHS Foundation Trust:* A Hutchison, A Asari, D Waring, R Sajith, H Walton; *Morriston Hospital, Swansea:* A Mikhail, I Hilldrup, F Latif, R Shrivastava; *New Cross Hospital, The Royal Wolverhampton NHS Trust:* J Nicholas, T Byrne, KS Sandhu, BK Tan, J Ward, D Whistance-Smith; *Northern General Hospital, Sheffield Teaching Hospitals NHS Foundation Trust:* A Khwaja, Y Jackson, B Kawar, J Middle, J Sorrell; *Nottingham City Hospital, Nottingham University Hospitals NHS Trust:* C Byrne, S Brand, E McHaffie; *Oxford University Hospitals NHS Foundation Trust:* R Haynes, S Crosbie, PK Judge, K Lafferty, S Ruse, E Sharples, BC Storey; *Queen Alexandra Hospital, Portsmouth Hospitals NHS Trust:* A Sampson, A Kirk, R Lewis, L Vinall, L Watkins, F Williams; *Queen Elizabeth Hospital, University Hospitals Birmingham NHS Foundation Trust:* P Cockwell, N Anderson, M Dutton, C Ferro, L Fifer, S Smith, S Stringer; *Royal Berkshire Hospital:* N Bhandary, B Alchi, J Foxton, L Jones; *Royal Devon & Exeter NHS Foundation Trust:* R D'Souza, D Harrison, S Heddon; *Royal Free Hospital NHS Foundation Trust:* DC Wheeler, B Caplin, E Damian, T Sobande; *Royal Stoke University Hospital, University Hospitals of North Midlands:* S Davies, C Edwards, C Matthews, M Plaza, S Reddy; *Salford Royal NHS Foundation Trust:* PA Kalra, D Chiu, L Haydock, M Kershaw, C Wilson, D Vassallo; *Southmead Hospital, North Bristol NHS Trust:* S Methven, J Bowles, E Collins, S Dawson, A Power; *The James Cook University Hospital, South Tees Hospitals NHS Foundation Trust:* S Kardasz, C Laven, C Wroe; *The Princess Royal Hospital, Telford:* K Eardley, D Donaldson, L Tonks; *University Hospital Coventry:* W Ayub, G Evans, S Hewins; *University Hospital of Wales, Cardiff & Vale University Health Board:* D Fraser, J Bagshawe, D Foxwell, A Williams; *York Teaching Hospital NHS Foundation Trust:* D Border, S Birch, L Griffiths, C Jones, K McCullough, R Molyneux, D Richardson

**Steering Committee**

*Chairman* C Baigent *Principal Investigators* R Haynes (Clinical Coordinator), MJ Landray *Administrative coordinator* R Dayanandan *Computing* A Baxter *Statistics* N Staplin *Other members* A Bethel, L Bowman, N Brunskill, P Cockwell, WG Herrington, M Hill, PK Judge, PA Kalra, C Knott, JJ McMurray, K Murphy, M Taal, DC Wheeler.

**Data Monitoring Committee**

*Chairman* K Wheatley *Statistician* J Emberson *Other members* C Tomson, P Roderick

**Randomized multicentre pilot study of sacubitril/valsartan versus irbesartan**

**in patients with chronic kidney disease:**

**UK Heart and Renal Protection (HARP)-III**

**Data Analysis Plan**

Contents

[1. Introduction 6](#_Toc450207617)

[2. Outcomes in UK HARP-III 6](#_Toc450207618)

[2.1 Primary outcome 6](#_Toc450207619)

[2.2 Secondary outcomes 6](#_Toc450207620)

[2.3 Tertiary outcomes 6](#_Toc450207621)

[3. Baseline characteristics 7](#_Toc450207622)

[4. Comparisons of sacubitril/valsartan versus irbesartan 8](#_Toc450207623)

[4.1 Primary assessment 8](#_Toc450207624)

[4.2 Secondary assessments of the primary outcome 8](#_Toc450207625)

[4.3 Assessment of secondary outcomes 9](#_Toc450207626)

[4.4 Assessment of tertiary outcomes 9](#_Toc450207627)

[5. Safety and tolerability outcomes 10](#_Toc450207628)

[5.1 Serious adverse events (SAEs) 10](#_Toc450207629)

[5.2 Reported reasons for stopping study treatment 10](#_Toc450207630)

[5.3 Biochemical safety data 11](#_Toc450207631)

[6. Details of analyses 11](#_Toc450207632)

[6.1 Methods of analysis 11](#_Toc450207633)

[6.1.1 ANCOVA 11](#_Toc450207634)

[6.1.2 Repeated measures 12](#_Toc450207635)

[6.1.3 Imputation of missing data 12](#_Toc450207636)

[6.1.4 Pharmacokinetic assessments 13](#_Toc450207637)

[6.1.5 Safety analyses 14](#_Toc450207638)

[6.2 Allowance for multiplicity of comparisons 14](#_Toc450207639)

[6.3 Tests for heterogeneity 15](#_Toc450207640)

[7. References 16](#_Toc450207641)

# Introduction

The purpose of this Data Analysis Plan is to provide a clear definition of the main randomized analyses to be reported in the primary report of the UK HARP-III trial results, before unblinding of the treatment allocation. The nature of further analyses and the content of subsequent publications cannot be specified in detail but, where appropriate, the general analytical approach is set out.

# Outcomes in UK HARP-III

## Primary outcome

The primary outcome is mean measured glomerular filtration rate (mGFR; adjusted for body-surface area) at 12 months. Glomerular filtration rate (GFR) will be measured using a ^51^Cr-EDTA or other approved technique.

## Secondary outcomes

The secondary outcomes are:

- Mean urine albumin:creatinine ratio [uACR] at 3, 6 and 12 months from centrally analysed urine samples.
- Estimated GFR [eGFR] at 3, 6 and 12 months from centrally analyses plasma samples using CKD-EPI formula.
- Metabolites of sacubitril/valsartan measured in blood samples taken at 3 months.

## Tertiary outcomes

The tertiary outcomes are:

- Systolic and diastolic blood pressure (mmHg) at 1, 3, 6, 9 and 12 months
- Markers of renal damage (kidney injury molecule-1 [KIM-1] and neutrophil gelatinase-associated lipocalin [NGAL]) at 6 and 12 months
- Markers of renal tubular function (β_2_-microglobulin and retinol binding protein) at 6 and 12 months
- Urine cyclic guanosine monophosphate (cGMP) excretion at 6 and 12 months
- Cardiac biomarkers (troponin I and N-terminal prohormone brain natriuretic peptide [NT-proBNP]) at 6 and 12 months
- Rate of change of eGFR calculated from creatinine values at Randomization, 1, 3, 6, 9 and 12 months (overall, and separately for 0-3 months [ie, Randomization, 1 and 3 month values] and 3-12 months [ie, 3, 6, 9 and 12 month values]) using the Chronic Kidney Disease Epidemiology Collaboration (CKD-EPI) formula. Where values from the central laboratory are available (randomization, 3, 6 and 12 months) these will be used, but local values will be used at 1 and 9 months.

# Baseline characteristics

In order to assess balance of baseline characteristics between randomized arms, the following variables recorded at randomization will be presented for each of the sacubitril/valsartan and irbesartan groups:

- Age
- Sex
- Past medical history (prior diabetes mellitus, prior vascular disease)
- Blood pressure (systolic and diastolic separately)
- Body mass index
- Baseline mGFR
- Baseline albuminuria
- Baseline 24 hour urinary sodium excretion (top versus bottom half)
- Current/recent medication (including any renin-angiotensin system [RAS] blockade)
- Cause of kidney disease (glomerular, tubulointerstitial, diabetic, hypertensive/renovascular, other systemic diseases, familial/hereditary, other known causes and unknown cause)

# Comparisons of sacubitril/valsartan versus irbesartan

All comparisons will involve comparing outcomes during the scheduled treatment period among *all* those participants allocated at randomization to receive sacubitril/valsartan 97/103mg twice daily versus all those allocated to receive irbesartan 300mg once daily (i.e. “intention-to-treat” [ITT] analyses).^1, 2^

## Primary assessment

Mean mGFR at 12 months will be compared between all participants allocated sacubitril/valsartan and all participants allocated irbesartan. Estimates will be made by analysis of covariance (ANCOVA) after adjustment for each participant’s baseline mGFR. Missing or implausible mGFR values will be handled as described in section 6.1.3.

## Secondary assessments of the primary outcome

Mean mGFR at 12 months (the primary outcome) among sacubitril/valsartan-allocated and irbesartan-allocated participants will be compared separately by the following baseline characteristics:

- Age (≤60; >60 years)
- Sex (Female, Male)
- History of diabetes mellitus (Yes, No)
- History of vascular disease (Yes, No)
- Systolic blood pressure (≤140; >140 mmHg)
- Diastolic blood pressure (≤80; >80 mmHg)
- Body mass index (top versus bottom half)
- Baseline mGFR (≤45; >45 mL/min/1.73m^2^)
- Baseline uACR (≤30 mg/mmol; >30 mg/mmol)
- Baseline 24 hour urinary sodium excretion (top versus bottom half, ignoring participants with missing values)
- Use of RAS blockade at screening (Yes, No)
- Cause of kidney disease (in categories as in Section 3)

## Assessment of secondary outcomes

Mean uACR (or an appropriate transformation of uACR) at 3, 6 and 12 months will be compared between all participants allocated sacubitril/valsartan and all participants allocated irbesartan using ANCOVA to adjust for baseline uACR. This will be done both separately at the three follow-up time points and overall (using the mean of the 3, 6 and 12 month values).

Pharmacokinetic analyses will also be conducted using measurements of sacubitril/valsartan metabolite trough concentrations measured at 3 months after randomization (see section 6.1.4).

## Assessment of tertiary outcomes

Mean values of tertiary outcomes (or mean of an appropriate transformation of the outcome) at 12 months will be compared between all participants allocated sacubitril/valsartan versus all patients allocated irbesartan. ANCOVA will be used to estimate the mean value adjusted for the baseline value. For tertiary outcomes that are also measured at 1, 3, 6 and 9 months (systolic and diastolic blood pressure and eGFR), analyses will also be done separately for the 1, 3, 6 and 9 month follow-up visits, as well as overall (using a weighted average of the 1, 3, 6, 9 and 12 month values).

Any other comparisons of the tertiary outcomes will also be between all participants allocated sacubitril/valsartan versus all participants allocated irbesartan but will be exploratory only (with due allowance in the interpretation for multiplicity and the retrospective nature of such analyses).

# Safety and tolerability outcomes

The safety and tolerability of sacubitril/valsartan will be assessed from the following information. The analyses of these data are described in section 6.1.5.

## Serious adverse events (SAEs)

All SAEs, regardless of whether the SAE is considered related to study treatment, will be recorded, and subdivided by outcome (fatal/non-fatal). The numbers and proportions of participants with SAEs in each group (sacubitril/valsartan and irbesartan) will be described. Particular SAEs of interest include:

- Angioedema
- Hypotension
- Need for dialysis (recorded on electronic case report form)

## Reported reasons for stopping study treatment

All reasons for stopping treatment will be recorded and listed in relevant categories by treatment allocation. All adverse events, including non-serious adverse events, that cause participants to discontinue study treatment will be recorded and grouped by treatment allocation according to MedDRA version 14.0 primary system organ class. The numbers and proportions of participants in each treatment group with non-serious adverse events and serious adverse events that result in discontinuation of study treatment will be described. In particular, discontinuation of study treatment due to the following reasons is of interest:

- Angioedema
- Hypotension
- Hyperkalaemia
- Deterioration in renal function
- Abnormal liver function tests (alanine aminotransferase [ALT] and aspartate aminotransferase [AST])

## Biochemical safety data

The biochemical safety collected will include kidney and liver related outcomes, in particular:

- Potassium (≥5.5 <6.0; ≥6.0 <6.5; ≥6.5 mmol/L)
- 25% reduction in eGFR since randomization
- ALT/AST >10x upper limit of normal (ULN)
- ALT/AST >3x ULN and bilirubin ≥2x ULN
- Consecutive ALT/AST >3x ULN (ie, two consecutive measurements at least 3 days apart)

# Details of analyses

## Methods of analysis

### ANCOVA

Comparisons of continuous outcomes (eg, mGFR, uACR, systolic and diastolic blood pressure and any other biomarkers or physical measurements) between the allocated treatment arms will be performed using ANCOVA adjusted for each patient’s value at randomization.^3^ If continuous outcomes are not normally distributed then appropriate transformations (e.g. log transformation) will be made.

### Repeated measures

Where more than one follow-up value of a biomarker is available, comparisons of the mean values of the biomarker will be conducted at each follow-up time using ANCOVA adjusted for each participant’s baseline value of the biomarker. In addition, a weighted average of all the follow-up values (with weights proportional to the amount of time between visits) will be calculated for each participant and the mean values compared using ANCOVA adjusted for each participant’s baseline value.

### Imputation of missing data

All analyses will be done according to the intention-to-treat principle and hence, where missing, primary and secondary outcome data will be imputed. For each of the continuous outcomes (eg, mGFR, uACR) missing post-randomization results will be imputed using multiple imputation, using 20 imputed data sets, with results across imputations being combined using the methods of Rubin.^4^ The imputation procedure will take into consideration each participant’s key baseline characteristics (listed in section 3), treatment allocation and any intermediate follow-up values of the biomarker, where available. For patients who commence chronic dialysis during the study and for whom it is not possible to measure GFR at study end, a value of 0 will be imputed for the final mGFR. Values will be imputed for patients who die prior to their second mGFR. The results from these analyses will be compared with those from equivalent “complete-case” analyses, but primary emphasis will be placed on the results after multiple imputation. All multiple imputation analyses will be implemented using the multiple imputation procedure in SAS version 9.3 (SAS Institute, Cary NC), using the expectation-maximization algorithm (which assumes a multivariate normal distribution) to impute values. For any continuous variables with missing baseline values, the mean among those with observed values will be imputed.

#### Participants who refused consent for 12 months follow-up

A small number of participants who had been randomized before the protocol was amended to extend follow-up from 6 to 12 months refused consent for 12 months follow-up. These participants will have a mGFR performed at 6 months. Multiple imputation will be used to impute 12 month mGFR values for these participants, including all available information on GFR at 6 months in the imputation model.

#### Implausible mGFR values

Technical issues can cause GFR measurements to give spurious results, but this is typically not apparent until after the participant has already been randomized and started their randomized allocation (or stopped taking study treatment at the end of the trial). The differences between each mGFR value and its corresponding creatinine-based eGFR value (ie, the value based on a blood sample taken at the same timepoint as the mGFR) will be calculated, and the distribution of these differences inspected before any unblinded analyses are performed. Based on this inspection a threshold will be determined (eg, 95 or 99% centiles) such that any values that fall outside this threshold are ignored. Multiple imputation will be used to handle any missing values of mGFR generated.

### Pharmacokinetic assessments

The objective of pharmacokinetic analyses is to quantify the determinants of plasma concentrations of metabolites of sacubitril/valsartan (including LBQ657 [sacubitrilat], the active metabolite of sacubitril).

A single trough plasma sample for pharmacokinetic analysis is collected at the 3 month visit (including time since last drug dosage). This will be sent to a third party laboratory (WuXi AppTec, Shanghai, China) for measurement of sacubitril, valsartan and LBQ657. Participants will be included in analyses from these analyses if they were allocated and taking sacubitril/valsartan at the 3 month visit and the plasma sample was taken between 10 to 16 hours after the last dose. Plasma concentrations of sacubitril, valsartan and LBQ657 will be tabulated by baseline mGFR (unadjusted for body surface area). In addition, appropriate population pharmacokinetic modelling techniques will be used to identify the determinants of the plasma concentration of each metabolite. The variables to be assessed will include baseline mGFR (unadjusted for body surface area), time since last dose, albuminuria, age, sex, body surface area and weight.

### Safety analyses

All participants randomized to sacubitril/valsartan will be compared with all participants randomized to irbesartan, regardless of whether a participant received all, some or none of their allocated treatment (ie, ITT).^1, 2^ A participant may contribute to more than one assessment if they have events of more than one type (e.g. non-fatal hypotension followed by angioedema).

For reasons for stopping and safety biochemical outcomes, the effect of allocated treatment on the number of randomized participants with at least 1 event will be compared using standard tests for differences in proportions.

For the time-to-event analyses of adverse events, the effect of allocated treatment will be evaluated using survival analytic methods on the time to first event during the entire study period. For each outcome, the log-rank method will be used to estimate the average event rate ratio comparing all those allocated sacubitril/valsartan with all those allocated irbesartan.^2^ Estimates of event rate ratios will be shown with 95% confidence intervals and their associated log-rank p-values. In all analyses, two-sided p-values (2P) <0.05 will be considered statistically significant (after any adjustment for multiple testing [see section 6.2]).

## Allowance for multiplicity of comparisons

The primary outcome will be assessed without adjustment for multiplicity. For secondary and particularly the tertiary and exploratory analyses, allowance in their interpretation will be made for multiple hypothesis testing,^1, 2^ taking into account the nature of events (including timing, duration and severity) and evidence from other studies. In addition to the pre-specified comparisons, many other analyses will be performed with due allowance for their exploratory and, perhaps, data-dependent nature. Conventionally, two-sided P-values <0.05 are often described as “significant”. But, the larger the number of events on which a comparison is based and the more extreme the P-value (or, analogously, the further the confidence interval is from zero) after any allowance has been made for the nature of the particular comparison (i.e. primary, secondary or tertiary; pre-specified or exploratory), the more reliable the comparison and, hence, the more definite any finding will be considered.

## Tests for heterogeneity

When a number of different subgroups are considered, chance alone may lead to there being no apparent effect in several subgroups in which the effect of treatment really is about the same as is observed overall. In such circumstances, “lack of direct evidence of benefit” is not good “evidence of lack of benefit”, and clearly significant overall results would provide strong indirect evidence of benefit in some small subgroups where the results, considered in isolation, are not conventionally significant (or, even, perhaps, slightly adverse).^1, 2^ Hence, unless the proportional effect in some specific subcategory is clearly different from that observed overall, the effect in that subcategory is likely to be best estimated indirectly by applying the proportional effect observed among all patients in the trial to the absolute risk of the event observed among control patients in that category.

Tests for heterogeneity of the proportional effect observed in subgroups will be used (with allowance for multiple comparisons) to determine whether the proportional effects in specific subcategories are clearly different from the overall effect.^1, 2^ If, however, three or more patient categories can be arranged in some meaningful order (e.g. age at randomization: <50, ≥50<60, ≥60) then assessment of any trend will be made. For subgroups based on continuous variables (e.g. blood pressure, kidney function), approximate similar sized divisions (such as by tertiles) may be used, using natural breaks to define categories (e.g. systolic blood pressure <140 mmHg rather than <138.7 mmHg). These breaks will be defined exactly prior to any unblinding of results.

# References

1. Peto R, Pike MC, Armitage P, et al. Design and analysis of randomized clinical trials requiring prolonged observation of each patient. I. Introduction and design. *Brit J Cancer* 1976; 34: 585-612.

2. Peto R, Pike MC, Armitage P, et al. Design and analysis of randomized clinical trials requiring prolonged observation of each patient. II. analysis and examples. *Brit J Cancer* 1977; 35: 1-39.

3. Vickers AJ, Altman DG. Statistics notes: Analysing controlled trials with baseline and follow up measurements. *Brit Med J* 2001; 323: 1123-4.

4. Rubin D. Multiple imputation for non-response in surveys. New York: John Wiley; 1987.

**Safety monitoring procedures**

1. **Abnormal potassium**
   1. *Potassium >5.5 <6.0 mmol/L*

- Confirm potassium in non-haemolyzed sample
- Inform Local Lead Investigator (LLI) within 72 hours
- Dietary advice to avoid potassium-rich food and drink
- Review non-study medications (including over-the-counter medications) and stop potassium-sparing medications if possible
- Consider checking for acidosis and correcting if present
- Repeat potassium measurement within 7 days
  - If potassium remains >5.5 <6.0 mmol/L, discuss frequency of potassium monitoring with LLI (and coordinating centre if required)
  - If potassium ≤5.5 mmol/L, return to routine follow-up
  1. *Potassium ≥6.0 mmol/L*
- Confirm potassium in non-haemolyzed sample
- Inform LLI immediately
- Discontinue study treatments
- Dietary advice to avoid potassium-rich food and drink
- Review non-study medications (including over-the-counter medications) and stop potassium-sparing medications if possible
- Consider checking for acidosis and correcting if present
- Repeat potassium measurement within no more than 3 days (ideally 1-2 days if potassium >6.5 mmol/L)
  - If <6.0 mmol/L, restart study treatment and discuss frequency of potassium monitoring with LLI (and coordinating centre if required)
  - If ≥6.0 mmol/L remain off all study treatment permanently

1. **Unexpected changes in estimated glomerular filtration rate (eGFR)**

If the eGFR falls by more than 25% between study visits (in particular, during the titration period in the first month after randomization) the LLI should be informed so that alternative causes of deterioration may be investigated (e.g. hypovolaemia, obstruction, non-study medications). The eGFR should be re-measured within 7 days (fewer days if a larger fall) in case of measurement error. If necessary the dose of study treatment can be modified, ideally after discussion with the coordinating centre.

1. **Abnormal alanine aminotransferase (ALT) or**  **aspartate aminotransferase (AST)**

| **ALT or AST (xULN)** | **Symptoms^[[1]](#footnote-1)^/bilirubin** | **Action** | **Follow-up monitoring** |
| --- | --- | --- | --- |
| ≤3 | Not relevant | None | Repeat at next study visit |
| >3 ≤5 | Absent and  Bilirubin <2x ULN | Inform LLI  Repeat in 1 week: investigate for cause^**^ if still >3 | At LLI’s discretion |
| >5 ≤8 | Absent and  Bilirubin <2x ULN | Inform LLI  Repeat in 2-4 days: investigate for cause^**^ if still >3 | Repeat ALT/AST, ALP and bilirubin until resolution (frequency at LLI’s discretion)  If >5 for >2 weeks, stop study treatment |
| >8 | Absent and  Bilirubin <2x ULN | Inform LLI  Repeat in 2-4 days: if still >8, stop study treatment.  Investigate for cause^**^ | Repeat ALT/AST, ALP and bilirubin until resolution (frequency at LLI’s discretion)  If >5 for >2 weeks, stop study treatment |
| >3 | Present or  Bilirubin ≥2x ULN | Inform LLI  Stop study treatment  Investigate for cause^**^ | Repeat ALT/AST, ALP and bilirubin until resolution (frequency at LLI’s discretion)  Consider restart if alternative cause found |

ULN = upper limit of normal; LLI = local lead investigator;

**Substantial amendments to the study protocol**

| **Version** | **Date** | **Original text** | **Amended text** | **Rationale** |
| --- | --- | --- | --- | --- |
| 5.0 | 04/09/2014 | Version approved by ethics committee and regulatory agency prior to recruitment beginning |  |  |
| 5.1 | 19/11/2014 |  | First morning void urine samples will be collected at each study visit for local and central analysis | First morning urine samples reduce intra-individual variability compared with random urine samples |
| 6.0 | 09/03/2015 | Inclusion criteria:  eGFR ≥20 <60 mL/min/1.73m^2^ and urine albumin:creatinine ratio >20 mg/mmol  Exclusion criteria:  Serum potassium > 5.2 mmol/L  Systolic BP <130 mmHg at Randomization | Inclusion criteria:  eGFR ≥20 <45 mL/min/1.73m^2^; or  eGFR ≥45 <60 mL/min/1.73m^2^ and urine albumin:creatinine ratio >20 mg/mmol  Exclusion criteria:  Serum potassium > 5.5 mmol/L  Systolic BP <110 mmHg (or <130 mmHg with symptoms of orthostatic hypotension) at Randomization | To facilitate recruitment and avoid unnecessary exclusion of participants |
| 7.0 | 11/05/2015 | Follow-up duration 6 months | Follow-up duration 12 months | New data from heart failure population suggested that the full effects on renal function may take at least 9 months to emerge with sacubitril/valsartan^1^ |
| 8.0 | 25/01/2016 | Original sample size 360 participants (based on assumption that 10% might discontinue study treatment) | Sample size increased to at least 400 participants | To allow for up to 15% of participants to discontinue study treatment |

1. Voors AA, Gori M, Liu LC, et al. Renal effects of the angiotensin receptor neprilysin inhibitor LCZ696 in patients with heart failure and preserved ejection fraction. *Eur J Heart Fail* 2015; 17: 510-7.

1. Symptoms of liver disease e.g. malaise, fatigue, abdominal pain, nausea, vomiting, jaundice

   ^**^ Careful history of alcohol, non-study medications, travel, diet, hepatobiliary ultrasound, viral and autoimmune serology [↑](#footnote-ref-1)
